# Supplementary material for: Identification and Evaluation of Novel Antigen Candidates against Salmonella Pullorum Infection Using Reverse Vaccinology
Source: Vaccines (Basel). 2023 Apr 18;11(4):865. doi: 10.3390/vaccines11040865 (PMC10143441; doi:10.3390/vaccines11040865)
Supplement: Supplementary file 1 [file vaccines-11-00865-s001.zip › Supplement Figures.pdf]

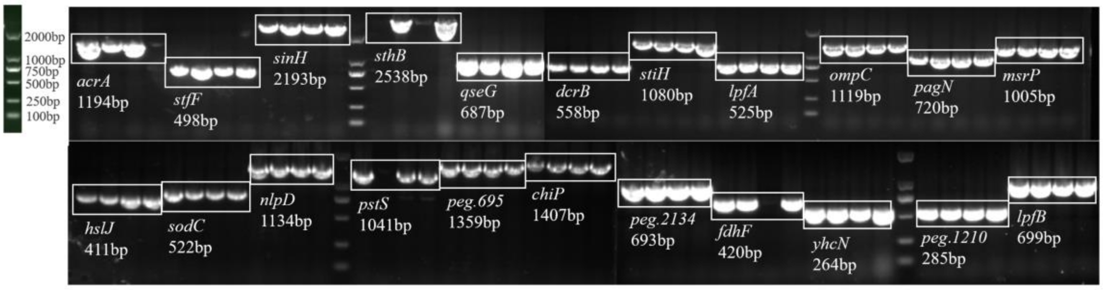

Supplementary Figure S1. Identification of 22 candidate vaccine target genes in different strains of *E. coli* Rosetta

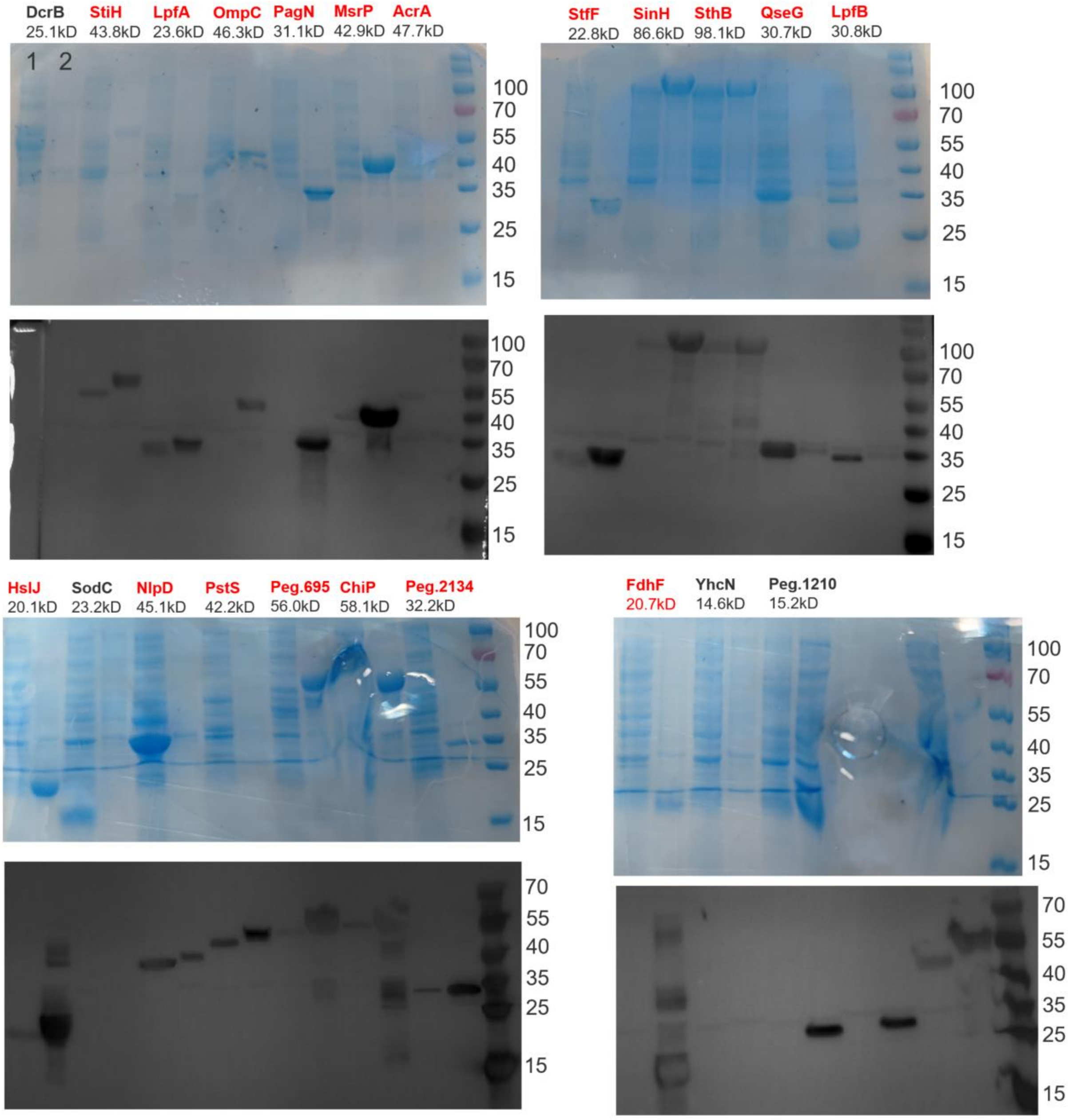

Supplementary Figure S2. SDS-PAGE and western blot analysis of 18 recombinant target proteins (red font) expressed in *E. coli* Rosetta. (1: Supernatant; 2: Inclusion Body)
